# Supplementary material for: Pre-COVID-19 ex vivo cross-reactive IFN-γ cellular response to SARS-CoV-2 spike overlapping peptides is more prevalent among Kenyan compared to Swedish adults
Source: BMC Infect Dis. 2026 Jan 17;26:174. doi: 10.1186/s12879-026-12582-6 (PMC12849388; doi:10.1186/s12879-026-12582-6)
Supplement: Supplementary file 1 — Supplementary Material 1 [file 12879_2026_12582_MOESM1_ESM.pdf]

Supplementary Materials

Pre-COVID-19 *ex vivo* cross-reactive IFN-γ cellular response to SARS-CoV-2 spike overlapping peptides is more prevalent among Kenyan compared to Swedish adults

Authors

Perpetual Wanjiku[1], Benedict Orindi[1], Jedidah Mwacharo[1], James Chemweno[1], Henry Kibe Karanja[1], Barbara Kronsteiner[2,3] Oscar Kai[1], Daniel Wright[4], Lynette Isabella Ochola-Oyier[1,5], Christopher Sundling[6,7], Susanna Dunachie[2,3], George M Warimwe[1,5], Anna Färnert[6,7], Philip Bejon[1,8], Francis M. Ndungu[1,5,6†]*, Eunice Nduati[1,5†].

Affiliations

1. Centre for Geographic Medicine Research (Coast), Kenya Medical Research Institute (KEMRI)-Wellcome Trust Research Programme, Kilifi, Kenya
2. Centre for Global Health Research, Nuffield Department of Medicine, University of Oxford, Oxford, United Kingdom
3. Mahidol-Oxford Tropical Medicine Research Unit, Mahidol University, Thailand
4. Department of Pediatrics, University of Oxford, Oxford, United Kingdom
5. Centre for Tropical Medicine and Global Health, Nuffield Department of Medicine, University of Oxford, Oxford, United Kingdom
6. Division of Infectious Diseases, Department of Medicine Solna, and Center for Molecular Medicine, Karolinska Institutet, Stockholm, Sweden
7. Department of Infectious Diseases, Karolinska University Hospital, Stockholm, Sweden
8. Modernising Medical Microbiology, Nuffield Department of Medicine, University of Oxford, Oxford, United Kingdom

* Correspondence to: fndungu@kemri-wellcome.org

†These authors contributed equally.

Table S1. Participants tested for each peptide

			Tested										
**Group**	**P1**	**P2**	**P3**	**P4**	**P5**	**P6**	**P7**	**P8**	**P9**	**P10**	**P11**	**P12**	**All participants**
**Kenyan pre-COVID-19 adults**	79	79	79	79	78	79	79	79	79	79	79	79	**79**
**Kenyan pre-COVID-19 infants**	3	3	10	10	3	10	3	10	3	10	3	4	**10**
**Swedish pre-COVID-19 adults**	7	6	18	18	6	18	7	18	6	15	6	5	**18**
**Kenyan COVID-19 adults**	18	19	37	36	15	37	23	33	17	31	15	14	**37**

Table S2. Participant demographic and clinical characteristics†.

Characteristic	Kenyan pre-COVID-19 adults (n = 79)	Kenyan pre-COVID-19 infants (n = 10)	Swedish pre-COVID-19 adults (n = 18)	Kenyan COVID-19 adults (n = 37)
**Median age (IQR), years**	31 (24, 40)	0.75 (0.58, 0.83)	33 (31, 41)	43 (34, 55)
**Missing age**	15	0	8	12
**Sex**				
**Female**	45 (57%)	5 (50%)	5 (28%)	17 (46%)
**Male**	23 (29%)	5 (50%)	1 (5%)	18 (49%)
**Missing (sex)**	11 (14%)	0	12 (67%)	2 (5%)

†Data are median (IQR) or number (%).

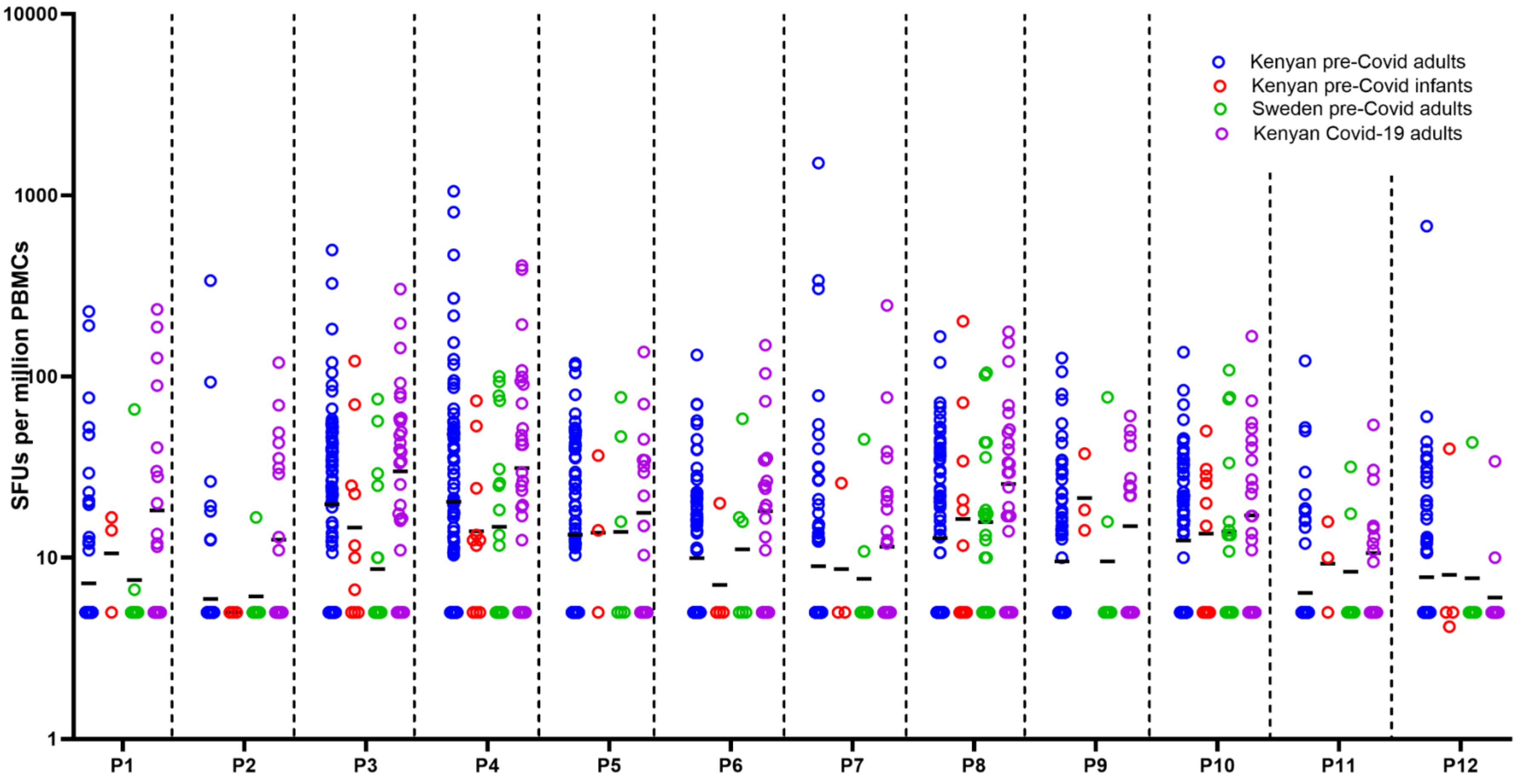

Figure S1. Frequencies of IFN-gamma secreting cell responses to individual but overlapping SARS-CoV-2 Spike peptides pools (P1-P12). Black line shows Geometric mean

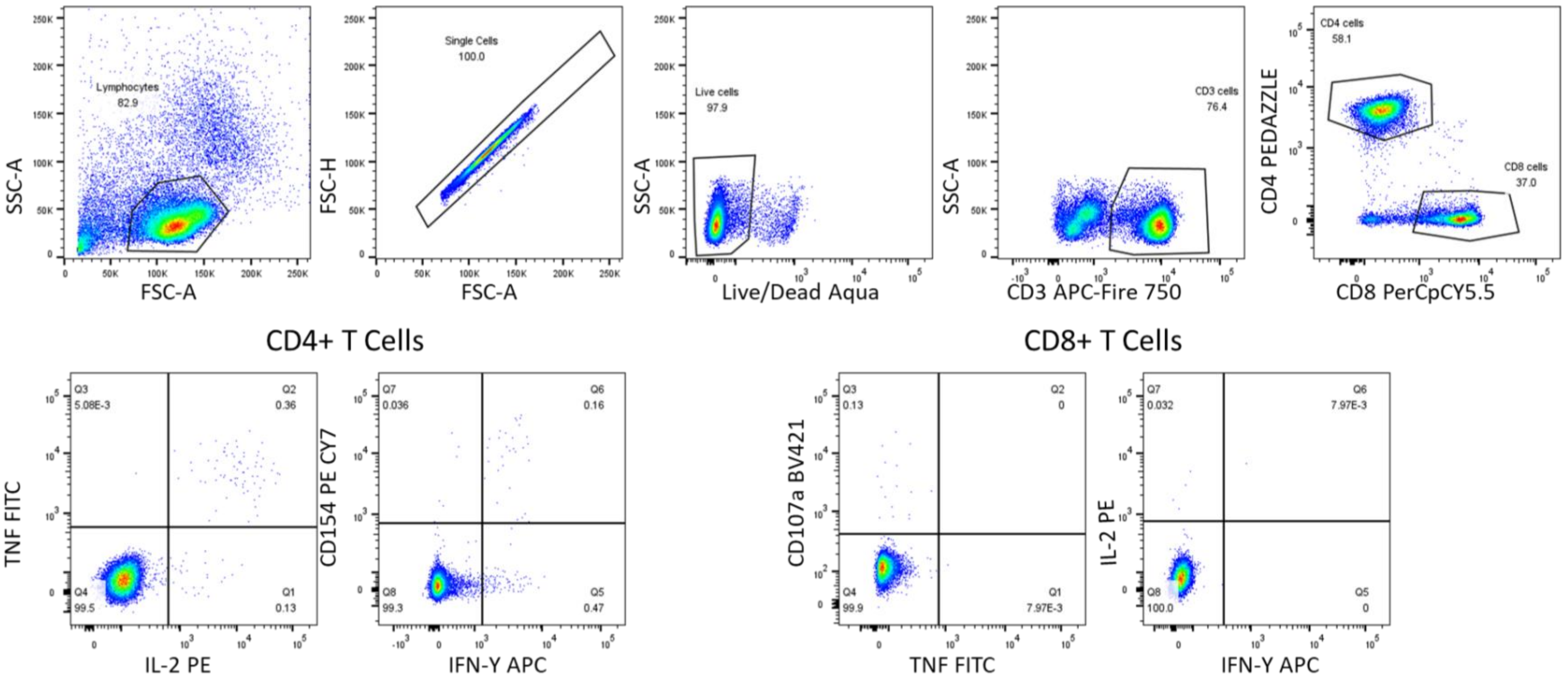

Figure S2. ICS gating strategy.

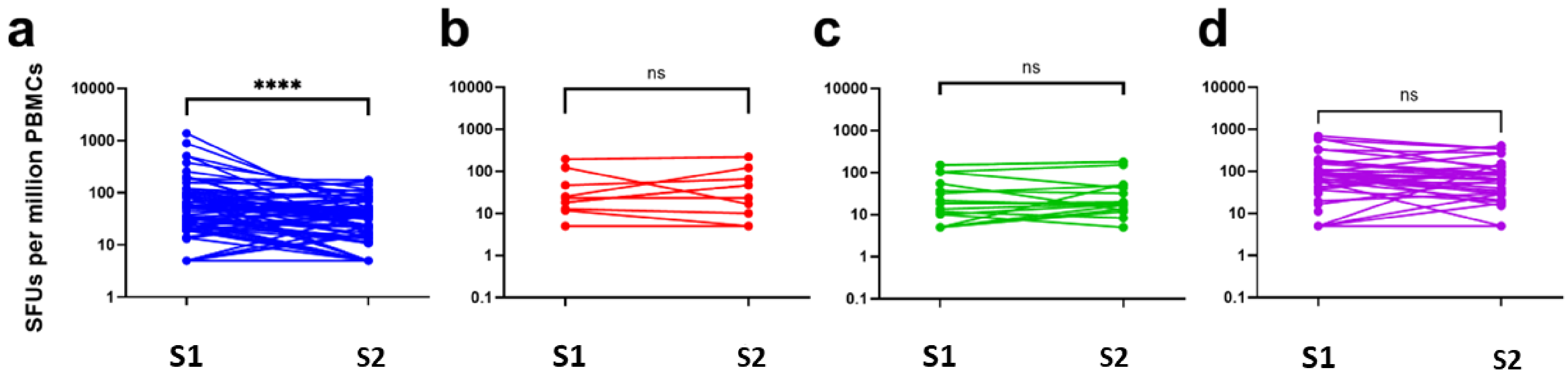

Figure S3. Interindividual variability of IFN-gamma secreting cell frequencies between SARS-CoV-2 Spike regions S1 and S2.

a Kenyan pre-COVID-19 adults samples, **b** Kenyan Pre-COVID-19 infants samples, **c** Sweden Pre-COVID-19 adults samples and **d** Kenyan COVID-19 adult patient samples. Comparisons were done using Wilcoxon matched pairs signed rank test. ****P < 0.0001

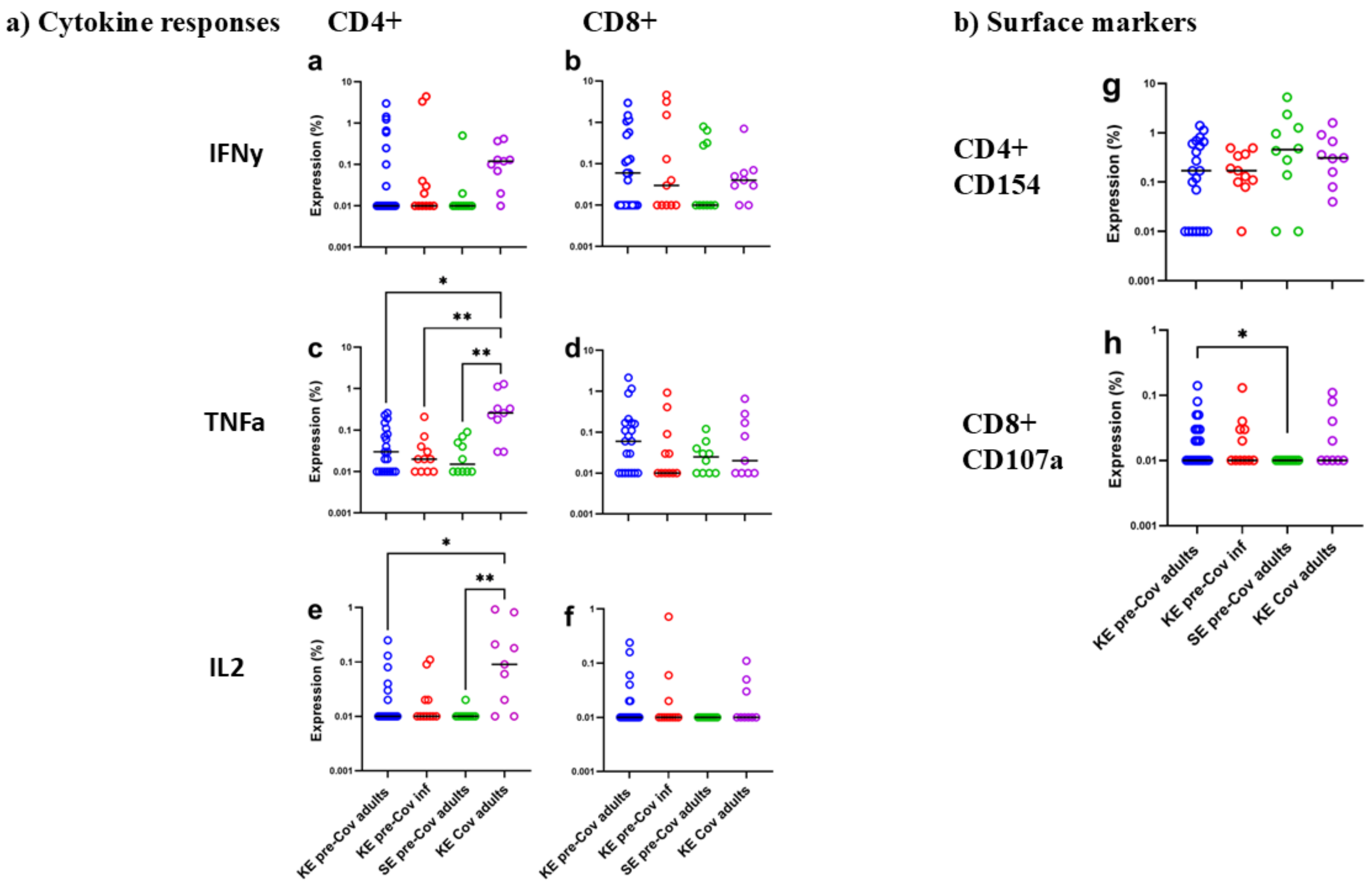

Figure S4. Proportions of T cells expressing cytokines and surface markers in response to SARS-CoV-2 Spike protein. **a) Cytokine responses** levels of **a** IFN-γ in CD4+ T cells, **b** IFN-γ in CD8+ T cells, **c** TNF in CD4+ T cells, **d** TNF in CD8+ T cells, **e** IL-2 in CD4+ T cells, **f** IL-2 in CD8+ T cells **b)**

Surface markers g CD154 on activated CD4+ T cells **h** CD107a on CD8+ T cells. Number of participants for: Kenyan pre-covid adults = 21, Kenyan pre-covid infants = 11, Sweden pre-covid adults = 10, Covid-19 adults = 9. he Mann–Whitney test and Kruskal–Wallis test with Dunn's multiple comparisons test were used to compare the proportions of cytokine-secreting T cells and T-cell activation markers between Kenyan and Swedish pre-COVID-19 adult samples and across the study groups, respectively. The black line shows the median. * $P < 0.05$, ** $P < 0.01$.

KE- pre-cov adults is Kenyan pre-COVID-19 adult samples, KE- pre-cov Inf is Kenyan pre-COVID-19 infant samples, SE- pre-cov adults is Swedish pre-COVID-19 adult samples, and KE-Cov adults is Kenyan COVID-19 adult samples.

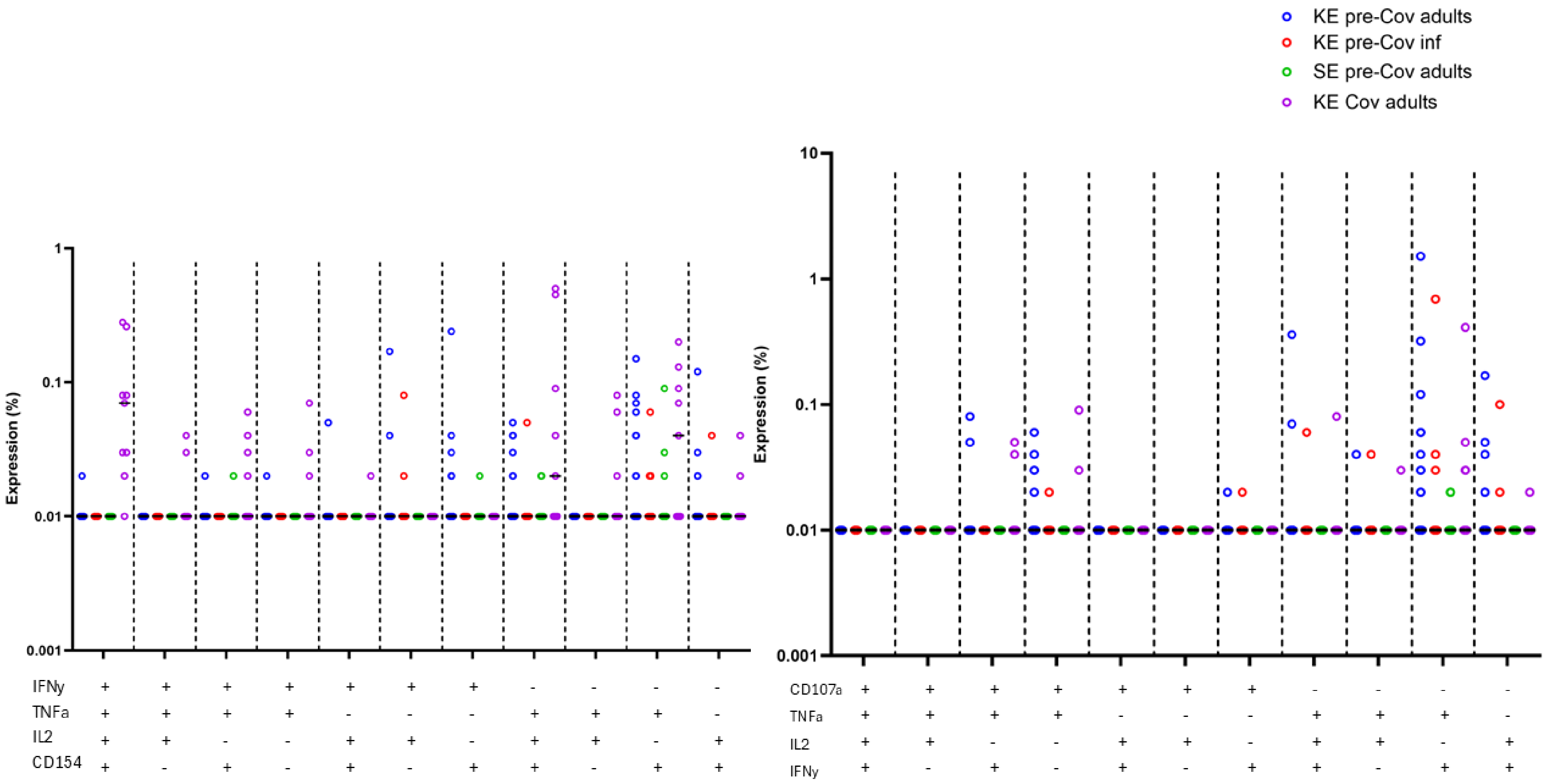

Figure S5a. Polyfunctional expression profile of CD4+ T cells. Figure S5b. Polyfunctional expression profile of CD8 + T cells.

Number of participants for: *Kenyan* pre-Covid adults samples = 21, *Kenyan* pre-covid infants samples = 11, Sweden pre-covid adults samples = 10, *Kenyan* Covid-19 adults samples = 9. Black line shows median. *KE- pre-cov adults is Kenyan pre-COVID-19 adult samples, KE- pre-cov Inf*

is Kenyan pre-COVID-19 infant samples, SE- pre-cov adults is Swedish pre-COVID-19 adult samples and KE-Cov adults is Kenyan COVID-19 adult samples.
